# In Vitro Assessment of the Efficacy of a Macrocyclic Chelator in Reversing Methylmercury Toxicity

**DOI:** 10.3390/ijerph16234817

**Published:** 2019-11-30

**Authors:** Paula Nobre, Maria de Fátima Cabral, Judite Costa, Margarida Castro-Caldas, Cristina Carvalho, Vasco Branco

**Affiliations:** 1 Research Institute for Medicines (iMed.ULisboa), Faculty of Pharmacy, Universidade de Lisboa, Av. Prof. Gama Pinto, 1649-003 Lisboa, Portugal; paulanobre@ff.ulisboa.pt (P.N.); fcabral@ff.ulisboa.pt (M.d.F.C.); jcosta@ff.ulisboa.pt (J.C.); mcastrocaldas@ff.ulisboa.pt (M.C.-C.); cristina.carvalho@ff.ulisboa.pt (C.C.); 2 UCIBIO, Departamento Ciências da Vida, Faculdade de Ciências e Tecnologia, Universidade NOVA de Lisboa, 2829-516 Caparica, Portugal

**Keywords:** methylmercury, chelators, thioredoxin reductase, thioredoxin, clinical toxicology

## Abstract

Methylmercury (MeHg) is a highly neurotoxic compound to which human populations are exposed via fish consumption. Once in cells, MeHg actively binds thiols and selenols, interfering with the activity of redox enzymes such as thioredoxin (Trx) and the selenoenzyme thioredoxin reductase (TrxR) which integrate the thioredoxin system. In fact, it has been shown that inhibition of this system by MeHg is a critical step in the unfolding of cell death. Current clinical approaches to mitigate the toxicity of MeHg rely on the use of chelators, such as meso-2,3-dimercaptosuccinic acid (DMSA) which largely replaced British anti-Lewisite or 2,3-dimercapto-1-propanol (BAL) as the prime choice. However, therapeutic efficacy is limited and therefore new therapeutic options are necessary. In this work, we evaluated the efficacy of a macrocyclic chelator, 1-thia-4,7,10,13-tetraazacyclopentadecane ([15]aneN_4_S), in preventing MeHg toxicity, namely by looking at the effects over relevant molecular targets, i.e., the thioredoxin system, using both purified enzyme solutions and cell experiments with human neuroblastoma cells (SH-SY5Y). Results showed that [15]aneN_4_S had a similar efficacy to DMSA and BAL in reversing the inhibition of MeHg over purified TrxR and Trx by looking at both the 5,5′-dithiobis(2-nitrobenzoic acid) (DTNB) reduction assay and insulin reduction capability. In experiments with cells, none of the chelating agents could reverse the inhibition of TrxR by MeHg, which corroborates the high affinity of MeHg to the selenol in TrxR active site. [15]aneN_4_S and BAL, unlike DMSA, could prevent inhibition of Trx, which allows the maintenance of downstream functions, although BAL showed higher toxicity to cells. Overall these findings highlight the potential of using [15]aneN_4_S in the treatment of MeHg poisoning and encourage further studies, namely in vivo.

## 1. Introduction

Methylmercury (MeHg) is reckoned as a highly neurotoxic compound, especially during fetal development when it can hinder neurodevelopment [1]. In extreme cases such as the mass poisoning event in Minamata, in utero exposure to very high levels of MeHg was shown to alter normal brain organization with severe consequences for the fetus, eventually leading to death [2]. 

In general, exposure of the human population to MeHg is much lower than in the case of the Minamata outbreak. However, populations consuming large amounts of fish—namely predatory species such as bluefin tuna, swordfish and black scabbard fish—can be exposed to MeHg levels that may cause cognitive and neuromotor deficits in children exposed in utero [3]. Therefore, pregnant and breast-feeding women should avoid consuming these species to diminish risk of neurodevelopmental effects in the newborn child [4,5]. 

The neurotoxic effects of MeHg encompass inhibition of glutamate re-uptake at the synaptic cleft [6] and changes to astrocyte reactivity [7]. On a mechanistic level, MeHg tightly binds reactive protein moieties such as thiol (SH–) and selenol (SeH–) groups in cysteine (Cys) and selenocysteine (Sec) residues, respectively [8,9,10]. Since these reactive groups are generally crucial for the activity of enzymes involved in redox regulation, exposure to MeHg results in loss of activity and function. In particular, the enzymes of the thioredoxin system are major targets for mercury compounds [11]. The thioredoxin system is responsible for the redox regulation of several cellular pathways, including DNA synthesis, apoptosis, cell signaling and antioxidant defense. It comprises thioredoxin (Trx)—a major dithiol-reducing enzyme—as well as the selenoenzyme thioredoxin reductase (TrxR) and β-nicotinamide adenine dinucleotide 2′-phosphate reduced tetrasodium salt hydrate (NADPH) [12,13].

Previously, we have shown in vitro and in vivo that Hg compounds including MeHg bind the active site of both Trx and TrxR resulting in inhibition of activity [10,14,15,16,17,18]. TrxR is particularly sensitive to MeHg due to the Sec residue in the open C-terminal region of the active site [14]. Moreover, we have shown that TrxR inhibition is an early event in neuronal cell death caused by Hg compounds. In fact, following inhibition of TrxR, Trx is kept functional by action of the glutathione/glutaredoxin system. When this backup mechanism is hampered, Trx becomes oxidized, triggering apoptotic cell death [18].

Limiting the consumption of contaminated fish is the most effective strategy to reduce exposure and the risk of methylmercury-related toxicity [5,19]. However, chelating agents arise as the main tool in clinical settings to remove MeHg from the body following intoxication events [20,21]. 

British anti-Lewisite or 2,3-dimercapto-1-propanol (BAL) was widely used in treating mercury intoxications but due to its toxicity has been replaced by water-soluble derivatives such as meso-2,3-dimercaptosuccinic acid (DMSA) or 2,3-dimercapto-1-propanesulfonic acid (DMPS) which are reported to be more effective than BAL in cases of MeHg poisoning with less secondary effects [22]. However, DMSA shows less affinity to mercurials than endogenous ligands such as glutathione (GSH) and is unable to cross cell membranes which limits its efficacy in removing Hg compounds from the brain [21]. Previous results from our group showed that BAL, DMSA and DMPS could protect TrxR from Hg^2+^ inhibition but had a more limited effect over MeHg, thus mimetizing clinical findings [14].

**Figure 1:**
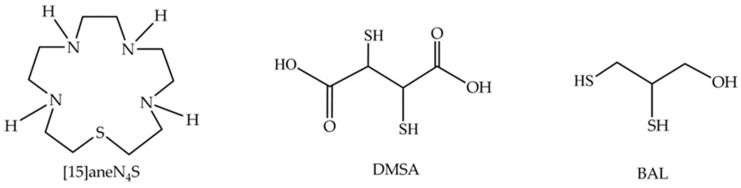
Chemical structures of the macrocycle 1-thia-4,7,10,13-tetraazacyclopentadecane ([15]aneN_4_S), meso-2,3-dimercaptosuccinic acid (DMSA) and 2,3-dimercapto-1-propanol (BAL).

Therefore, the development of new therapeutic options that may replace or complement existing ones is essential. In the last few decades, many macrocyclic chelators have been evaluated for clinical applications [23,24]. In fact, macrocyclic compounds may exhibit important properties such as high kinetic and thermodynamic stabilities and low toxicity, making them very promising agents in this context [25]. The macrocyclic chelator, 1-thia-4,7,10,13-tetraazacyclopentadecane ([15]aneN_4_S, Figure 1) was recently shown to have a high affinity for Hg^2+^ with a high thermodynamic stability constant (log *K*_ML_ = 23.74) indicating that this compound may be useful in chelation therapy [26]. 

Thus, this work aims to evaluate the efficacy of [15]aneN_4_S in reversing the toxicity of MeHg in comparison to BAL and DMSA, namely by looking at its ability to mitigate the inhibition of primary endogenous molecular targets like TrxR and Trx. For this purpose, we conducted assays with purified TrxR and Trx and with SH-SY5Y human neuroblastoma cells. 

## 2. Materials and Methods 

### 2.1. Reagents

Methylmercury chloride (CH_3_HgCl), dithiothreitol (DTT), 3-(4,5-dimethylthiazol-2-yl)-2,5-diphenyltetrazoliumbromide (MTT), 5,5′-dithiobis(2-nitrobenzoic acid) (DTNB), β-nicotinamide adenine dinucleotide 2′-phosphate reduced tetrasodium salt hydrate (NADPH), insulin (human recombinant), BAL and DMSA were purchased from Sigma-Aldrich. 

The macrocyclic compound [15]aneN_4_S was synthesized according to a previously described procedure [24] and the reactions reported in Figure S1 of the Supplementary Materials. The characterization was performed by NMR, FT-IR spectroscopy and electrospray ionization mass spectrometry (ESI-MS) (Figure S1 part B). Aqueous solution of [15]aneN_4_S was prepared at 2.2 mM, and its exact concentration was determined by potentiometry. 

Rat recombinant TrxR1 and human Trx1 were from IMCO, Sweden (www.imcocorp.se). Antibodies were all from Santa Cruz Biotechnologies, with the exception of the antibody for human thioredoxin 1 which was from IMCO.

Cell culture media and reagents were acquired from Gibco-ThermoFisher^®^. Fetal bovine serum was bought from Biochrome^®^ and inactivated at 56 ºC before use.

### 2.2. Determination of TrxR Activity by the DTNB Reduction Assay

Rat recombinant TrxR (20 nM) was incubated in 50 mM Tris-HCl, pH 7.5 at 37 ºC for 5 min with MeHg (200 nM), 100 µM NADPH and 40 µM of either [15]aneN_4_S, BAL or DMSA. The 40 µM level was chosen according to previous data from our group [14] and corresponds to the expected concentration in the human organism following a therapeutic dose of DMSA (30 mg/kg). To avoid stability issues, chelating agent solutions were freshly prepared prior to use.

TrxR activity was determined as previously described [27] by adding 100 µL of a DTNB solution (5 mM) in 50 mM of Tris, pH 7.5. Absorbance at 412 nm was followed for 5 min in a microplate reader and TrxR activity was obtained as the linear change in absorbance over the initial 2 min.

### 2.3. Trx System Activity Determination by Insulin Reduction Assay

To assess effects over the whole Trx system, activity was determined by a method previously described by Árner and Holmgren with modifications [27]. Briefly, 20 nM rat recombinant TrxR, 3 µM human Trx and NADPH (100 µM) were preincubated at 37 ºC for 5 min in 50 mM of Tris, pH 7.5 with MeHg (200 nM) and two different concentrations (1 and 40 µM) of either [15]aneN_4_S, BAL or DMSA. Afterwards, a master mix containing insulin and NADPH (final concentrations 160 and 200 µM, respectively) was added and the change in absorbance at 340 nm (NADPH consumption) was followed in a microplate reader. Activity was calculated as the linear change in absorbance over the initial 5 min.

### 2.4. Cell Culture

Human neuroblastoma cells (SH-SY5Y) were purchased from ATCC^®^ and cultured in a medium consisting of a 1:1 mixture of Dulbecco’s Modified Eagle’s Medium and F-12, supplemented with 10% fetal bovine serum and 1% Pen-Strep mixture in a humidified incubator at 37 °C and 5% CO_2_.

### 2.5. Cell Viability Assay

Cell viability was determined by the MTT assay after 24 h of exposure to each compound as described previously [15]. Briefly, cells (1 × 10^4^ cells/well) were cultured in 96-well plates (Nunc^®^) and allowed to seed for 24 h before addition of various concentrations of each chelating agent—[15]aneN_4_S (10, 20, 40, 80, 120 µM), BAL (10, 20, 40, 80, 120 µM), DMSA (10, 20, 40, 80, 120 µM). MTT was added to plates at a final concentration of 400 mg/mL followed by incubation at 37 ºC for 2 h. After incubation, the medium was removed and the formazan crystals were dissolved in a 4:1 DMSO/glycine buffer (pH 10.5) with shaking for 15 min at room temperature. Cell viability was then assessed by measuring formazan absorption at 550 nm in a microplate reader (Zenyth3100, Anthos Labtec Instruments). Appropriate controls for vehicle (ethanol 0.4%) and spontaneous MTT reduction by chelating agents were conducted in parallel.

Based on viability results, test concentrations for subsequent experiments were set at 40 µM for all chelating agents. The MeHg concentration used in the following experiments (2.5 µM) was selected based on previous results by our group [18].

### 2.6. Preparation of Cell Lysates

Cells were plated (1 × 10^6^ cells/dish) in culture dishes (100 mm × 2 mm) and allowed to reach 70%–80% confluence. Fresh medium was then supplied to cells followed by the addition of MeHg (2.5 µM) and chelating agents (40 µM final concentration). Coexposure to MeHg and each of the chelating agents was achieved by simultaneous addition to the culture media. All treatments lasted 24 h after which cells were harvested, washed in phosphate-buffered saline (PBS, pH 7.4) and centrifuged (600× *g*, 5 min) to obtain the final cell pellets which were disrupted in lysis buffer (25 mM Tris–Cl, pH 7.5; 100 mM NaCl; 2.5 mM EDTA; 2.5 mM EGTA; 20 mM NaF, 1 mM sodium orthovanadate, 20 mM sodium pyrophosphate; 20 mM sodium β-glycerophosphate; 0.5% TritonX-100 and protease inhibitor cocktail (Roche; 1 tablet per 10 mL)). Before activity determinations, samples were centrifuged at 12,000× *g* and 4 °C for 5 min. Total soluble protein and enzymatic activities were determined in the supernatant as described below.

### 2.7. Total Soluble Protein

Total protein was quantified using a modification of the Bradford Assay [28] by mixing each sample with 5× diluted Coomassie dye (Bio-Rad) in 96-well plates, followed by measurement of absorbance at 595 nm in a microplate reader. A calibration curve using BSA as a standard (0–16 µg/µL) was used to quantify protein levels.

### 2.8. TrxR and Trx Activity Determination

TrxR and Trx activities were determined with the insulin end-point assay described by Árner and Holmgren [27]. For TrxR activity, samples (40 µg of protein) were incubated in 96-well plates with 0.3 mM insulin, 660 mM NADPH, 3 mM EDTA and 2 µM Trx (IMCO Corp., Sweden)—previously reduced with dithiothreitol (DTT) and desalted in a NAP-5 column—in 85 mM HEPES buffer (pH 7.6) at 37 °C for 20 min. Control wells containing the same reagents excluding Trx addition were prepared in parallel. After the incubation period, 250 µL of a 1 mM DTNB solution in 6 M guanidine hydrochloride (pH 8.0) was added to each well and absorbance was measured in a microplate reader (Zenyth3100, Anthos Labtec Instruments) at 412 nm. TrxR activity was quantified as the difference in absorbance between the Trx-containing well and the control well. The determination of Trx activity followed the same procedure used for TrxR, but with the samples being incubated with 100 nM recombinant rat TrxR [27].

### 2.9. Expression of TrxR and Trx

Western blot was used to determine the effect of MeHg and chelating agents on the expression levels of TrxR and Trx. Samples (30 μg of protein) were separated under reducing conditions with SDS-PAGE on a 4%–12% Bis-Tris gel with MES running buffer (ThermoFisher^®^), followed by transfer to a nitrocellulose membrane which was subsequently blocked with a 5% skimmed milk solution and probed with the following primary antibodies: anti-human TrxR1 rabbit polyclonal IgG (sc-20147, Sta. Cruz), anti-human Trx1 rabbit polyclonal IgG (ATRX-03, IMCO Corp.), anti-human glyceraldehyde 3-phosphate dehydrogenase (GAPDH) rabbit polyclonal IgG (sc-25118, Sta. Cruz). Band intensity was quantified using the QuantityOne Software (Biorad^®^) and protein expression levels were normalized for protein loading on the gel, assessed either by Ponceau S staining prior to the blocking step or by quantification of GAPDH.

### 2.10. Statistical Analysis

Results in figures are presented as mean ± standard error (SE) (see Table S1 for details) of at least three independent experiments. Differences between groups were evaluated by the Mann–Whitney rank test considered significant at *p* < 0.05 and very significant at *p* < 0.01.

## 3. Results

### 3.1. Effect of MeHg and Chelating Agents on TrxR and Trx Activities

**Figure 2:**
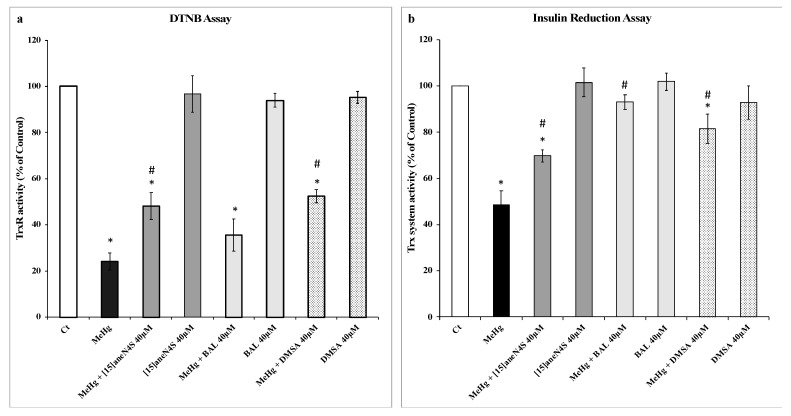
Activity of thioredoxin (Trx) system enzymes as measured by the 5,5′-dithiobis(2-nitrobenzoic acid) (DTNB) and insulin reduction assay. (**a**) Recovery of selenoenzyme thioredoxin reductase (TrxR, 20 nM) activity inhibited by methylmercury (MeHg, 200 nM) by the chelating agents [15]aneN_4_S, BAL and DMSA (40 µM) as measured by the DTNB reduction assay. (**b**) Recovery of Trx system activity by chelating agents ([15]aneN_4_S, BAL and DMSA; 40 µM) following incubation of TrxR (20 nM), Trx (3 µM) and β-nicotinamide adenine dinucleotide 2′-phosphate reduced tetrasodium salt hydrate (NADPH) with MeHg (200 nM) as measured by the insulin reduction assay. All data points represent the mean ± SE of at least three independent experiments. * Different from control, *p* < 0.05. # Different from the MeHg group, *p* < 0.05.

Exposure of rat recombinant TrxR (20 nM) to MeHg (200 nM) produced a very significant decrease in DTNB reduction (to 25% of control) (Figure 2a). None of the chelating agents caused a change in TrxR’s ability to reduce DTNB and all three were able to reduce the inhibitory effect of MeHg. Indeed, both [15]aneN_4_S and DMSA could recover TrxR activity to approximately 50% of control, whereas BAL could recover only to 36% of control.

In the insulin reduction assay (Figure 2b), incubation of the thioredoxin system enzymes with MeHg (200 nM) resulted in a drop in activity to 48% of control level. Similar to that observed with the DTNB assay, the chelating agents per se did not change insulin reduction levels significantly. In the test concentrations, chelating agents could recover Trx system activity for levels between 70% and 90% of control activity with efficacy for BAL > DMSA > [15]aneN_4_S (Figure 2b).

### 3.2. Chelating Agents and Cellular Viability 

**Figure 3:**
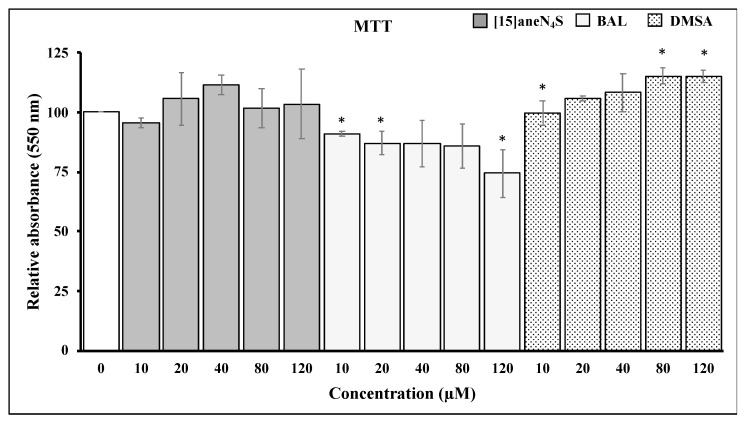
Changes in 3-(4,5-dimethylthiazol-2-yl)-2,5-diphenyltetrazoliumbromide (MTT) reduction ability by SH-SY5Y cells following exposure for 24 h to different concentrations of chelating agents. All data points represent the mean ± SE of at least three independent experiments. * Different relative to control, *p* < 0.05.

The MTT assay was used to assess the effect of the different chelating agents on cellular viability. Both [15]aneN_4_S and DMSA did not affect negatively the viability of cells. On the other hand, BAL caused a reduction of 25% in SH-SY5Y ability to reduce MTT following exposure to 120 µM for 24 h. However, this effect of BAL over cellular viability was already apparent at 10 µM (Figure 3). 

### 3.3. Thioredoxin Reductase and Thioredoxin Activity and Expression in SH-SY5Y Cells

**Figure 4:**
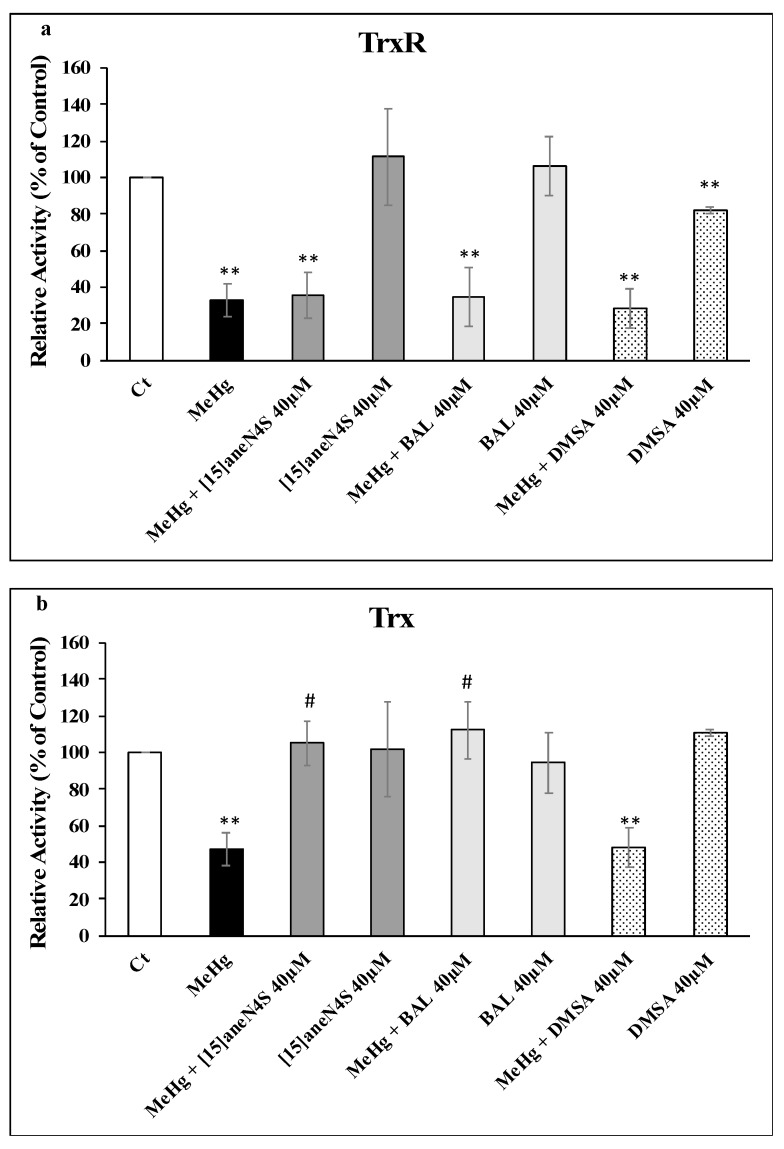
Activities of (**a**) TrxR and (**b**) Trx in SH-SY5Y cells exposed to MeHg and/or chelating agents for 24 h. All data points represent the mean ± SE of at least three independent experiments. Different relative to control: ** *p* < 0.01. # Different relative to MeHg exposure, *p* < 0.05.

Exposure of SH-SY5Y cells to 2.5 µM of MeHg for 24 h resulted in a decrease of TrxR activity to 33% of control level (*p* < 0.01). Exposure of cells to 40 µM of [15]aneN_4_S and BAL did not decrease TrxR activity, whereas DMSA caused a 20% drop in activity (*p* < 0.01). Most importantly, the chelating agents failed to prevent the inhibition of TrxR by MeHg (Figure 4a).

Thioredoxin activity was less affected by exposure of SH-SY5Y to MeHg with a drop to 47% of control (*p* < 0.01). Like with TrxR, the chelating agents did not affect Trx activity significantly. However, both [15]aneN_4_S and BAL were able to fully prevent Trx inhibition by MeHg (*p* < 0.05) whereas DMSA did not show any protective capability (*p* > 0.05) (Figure 4b). 

**Figure 5:**
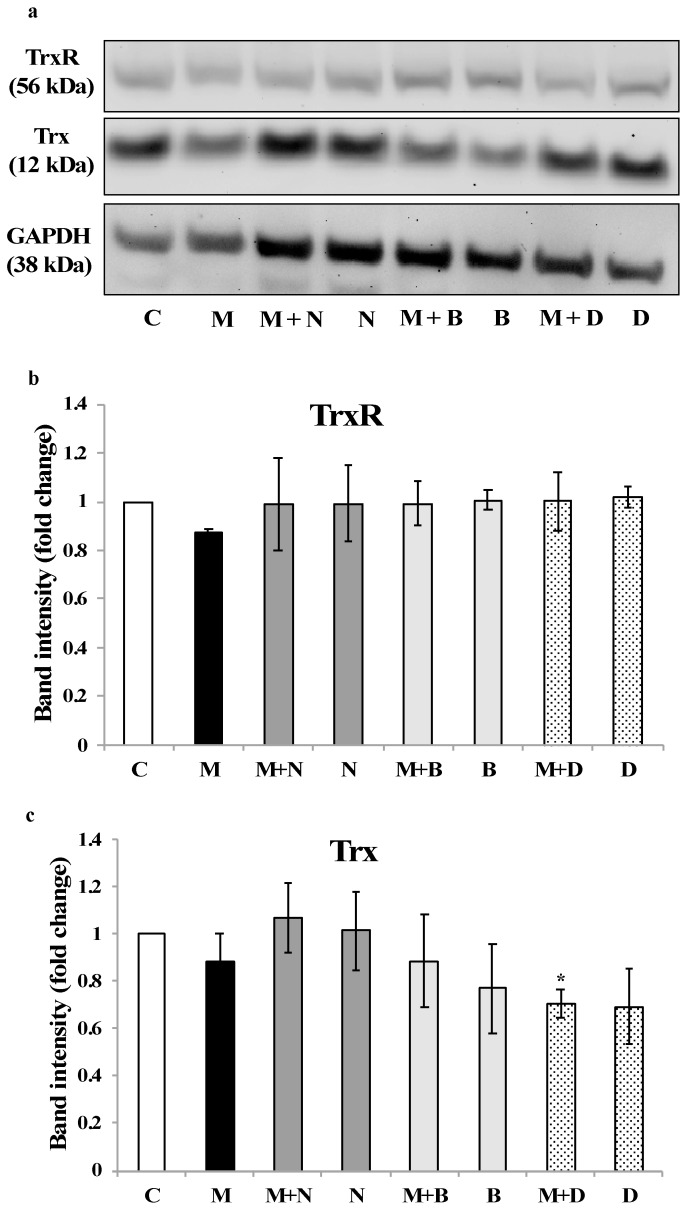
Expression of TrxR and Trx in SH-SY5Y cells exposed to MeHg and/or chelating agents for 24 h. (**a**) The Western blot images are representative of three independent experiments. Band intensity for (**b**) TrxR and (**c**) Trx was quantified in all replicates, normalized for GAPDH expression and plotted as fold-change relative to the control group. * Significantly different from control (*p* < 0.05). C—Control; M—MeHg; M + N—MeHg with coexposure to [15]aneN_4_S; N—[15]aneN_4_S; M + B—MeHg with coexposure to BAL; B—BAL; M + D—MeHg with coexposure to DMSA; D—DMSA.

Concerning the effect of each treatment on the expression of TrxR and Trx (Figure 5), it is interesting to note that none of the chelating agents altered the expression of TrxR (Figure 5a,b). On the other hand, Trx expression was down-regulated by DMSA, particularly in coexposure with MeHg (Figure 5c), which agrees with results concerning Trx activity.

## 4. Discussion

Mercury compounds are soft electrophiles that actively target protein thiols and selenols in Cys and Sec residues, respectively [6,11]. Selenols, due to their lower p*K*a (5.4), are deprotonated at physiological pH and are, therefore, preferential targets for Hg compounds such as MeHg. Indeed, TrxR is a known target for MeHg with its inhibition being a primary event in the unfolding of cell death, which involves subsequent oxidation of Trx and apoptosis initiation [18]. Therefore, an evaluation of the efficacy of chelating agents in preventing MeHg toxicity should take into account these relevant molecular targets. Previous experiments testing the efficacy of chelating agents (BAL, DMSA and DMPS) over TrxR inhibited by mercurials corroborated clinical findings, i.e., chelating agents showed a greater treatment efficiency for Hg^2+^ exposure relative to MeHg [14]. In this study we evaluated a promising chelating agent, the macrocycle [15]aneN_4_S, to assess if it had a better performance than traditional clinical antidotes in mitigating MeHg toxicity. 

Experiments with rat recombinant TrxR showed that MeHg produced a strong inhibition of TrxR activity, hindering its ability to reduce DTNB (Figure 2a), similar to former reports [10,14]. Also, similar to previous observations, inhibition of Trx system (Figure 2b) activity by MeHg in the insulin reduction assay was less exuberant (50%) than observed with the DTNB assay [10]. The insulin reduction assay involves a more complex molecular organization than the DTNB assay and is closer to what is found in biological systems (i.e., cells) where Trx is in excess over TrxR. Therefore, a redistribution of MeHg towards Trx or even insulin may justify the differences observed between the two assays. Nonetheless, both assays showed that all chelating agents could recover the activity of TrxR (DTNB assay) and/or the Trx system (insulin reduction assay) to a similar extent, which agrees with previous experiments using BAL, DMSA and DMPS [14]. In these assays chelating agents were in great molar excess over both MeHg (200-fold) and Trx system enzymes (13-fold over Trx) reflecting what is expected to be found following a therapeutic dosage. 

The efficiency of a chelating agent is directly linked to its ability to form stable complexes with the metal cation [21]. Thus, the fact that the recovery capability was not as effective in the DTNB assay, where only TrxR is present, probably means that binding of all three chelating agents to MeHg is more labile when compared to binding of MeHg with the selenol in TrxR’s active site, which at physiological pH (7.4) is deprotonated into a selenolate (Se^–^) and is therefore highly reactive with electrophiles [29]. 

This hypothesis was reinforced by the results in cell experiments—involving coexposure to chelating agents and a higher, physiologically relevant level of MeHg (2.5 µM)—where no protective effect could be observed over TrxR activity for any of the chelating agents (Figure 4a). Since no significant changes were observed in TrxR expression, the observed inhibition should be due to MeHg interaction with the active site of the enzyme (Figure 5). 

Most interestingly, Trx activity was fully protected from inhibition by MeHg during coexposure to either [15]aneN_4_S and BAL (Figure 4b). This shielding effect is of toxicological relevance since it allows Trx to secure essential functions for cell survival, such as antiapoptotic regulation of ASK-1 and reduction of ribonucleotide reductase which is essential for DNA synthesis. Indeed, although TrxR was inhibited by MeHg even in the cotreatment with the chelators, it is known that Trx can rely on backup mechanisms to maintain its turnover from the oxidized to the reduced state [18]. Nevertheless, BAL, although capable of effectively protecting cellular Trx from MeHg, showed negative effects over cellular viability already after 24 h of exposure (Figure 3), which confirms previous knowledge on its toxicity and limited therapeutic index [30]. Also, BAL knowingly increases brain deposition of MeHg in vivo which limits its long-term use. The chelating agent [15]aneN_4_S did not show any significant cytotoxicity in the concentration range tested (Figure 3).

Noteworthy is the fact that DMSA, a standard chelating agent in organic Hg poisoning [31], could not prevent Trx inhibition in cells exposed to MeHg and, actually, led to a decrease in Trx expression (Figure 5c). This is an interesting finding since DMSA is normally regarded as having a limited ability to cross cell membranes [27]. So, this result, together with the inhibitory effect over cellular TrxR activity (Figure 4a), indicates that DMSA, besides being ineffective in binding MeHg, has itself detrimental effects over the thioredoxin system [15].

## 5. Conclusions

In this study, we evaluated the efficacy of a macrocyclic chelator in preventing MeHg toxicity by comparison with standard clinical chelating agents, BAL and DMSA. A very relevant aspect of this study is the choice of toxicity endpoints that are primary molecular targets of MeHg, namely TrxR and Trx, which are involved in the mechanism of MeHg-induced cell death.

The chelator [15]aneN_4_S showed a capability comparable to that of BAL and DMSA in protecting the activity of purified suspensions of the thioredoxin system. In cells, [15]aneN_4_S proved less toxic than BAL and much more effective than DMSA in reversing effects over the thioredoxin system. Indeed, although experiments with cells revealed that none of the chelating agents were capable of protecting TrxR activity from MeHg, both [15]aneN_4_S and BAL could shield Trx activity against MeHg inhibition. Since the thioredoxin system is a highly relevant target in the unfolding of MeHg toxicity, this result encourages further research concerning the antidote capacity of [15]aneN_4_S, namely in vivo studies. This is especially relevant since the chelating agent [15]aneN_4_S presented a better compromise between efficacy and toxicity when compared with two other clinically relevant antidotes. 

## Supplementary Materials

The following are available online at https://www.mdpi.com/1660-4601/16/23/4817/s1, Figure S1: Synthesis and characterization of the macrocycle [15]aneN_4_S; Table S1: Mean ± Standard Error for each experimental group in Figure 2, Figure 3, Figure 4 and Figure 5.
