# Supplementary material for: In Vitro Assessment of the Efficacy of a Macrocyclic Chelator in Reversing Methylmercury Toxicity
Source: Int J Environ Res Public Health. 2019 Nov 30;16(23):4817. doi: 10.3390/ijerph16234817 (PMC6926914; doi:10.3390/ijerph16234817)
Supplement: Supplementary file 1 [file ijerph-16-04817-s001.pdf]

International Journal of
Environmental Research and Public Health

MDPI

Synthesis and characterization of the macrocycle [15]aneN$_4$S

A - Synthesis of the macrocycle [15]aneN$_4$S

The macrocycle 1-thia-4,7,10,13-tetraazacyclopentadecane ([15]aneN$_4$S) was prepared according to the reactions depicted in Figure 1. The first step involved the synthesis of the precursor diamide, 1-thia-4,7,10,13-tetraazacyclopentadecane-3,14-dione (dioxo-[15]aneN$_4$S) by reaction of the dimethyl ester of thiodiglycolic acid, with triethylenetetramine in dry methanol. The compound was purified by chromatography. Yield = 74%.

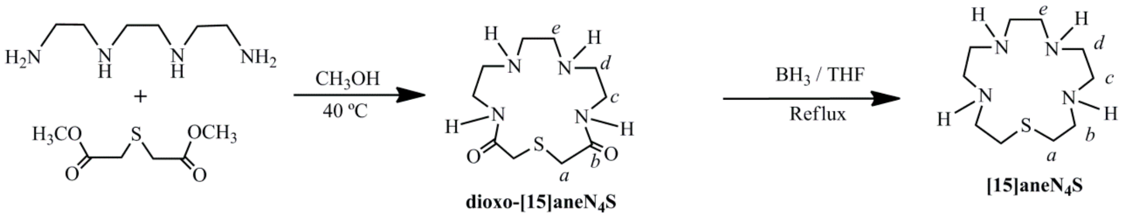

Figure S1. Schematic synthesis of [15]aneN$_4$S.

The reduction of the cyclic diamide dioxo-[15]aneN$_4$S with borane, in refluxing dry THF under nitrogen afforded the macrocycle [15]aneN$_4$S. The compound was purified by chromatography. Yield = 68%.

B - Characterization of the macrocycles dioxo-[15]aneN$_4$S and [15]aneN$_4$S

dioxo-[15]aneN$_4$S:

^{1}H NMR (400.13 MHz; D$_2$O; DSS; pD = 3.4): δ 3.26 (t, 4H, (triplet), H_d) 3.35 (s, 4H, (singlet), H_a), 3.53 (t, 4H, H_c), 3.57 (s, 4H, H_e) ppm.

^{13}C NMR (100.61 MHz; D$_2$O; dioxane; pD = 3.4): δ 35.3 (C_c), δ 37.5 (C_a), 42.8 (C_e), 48.2 (C_d), 175.0 (C_2) ppm.

FT-IR (KBr, cm^{-1}): ν 3427 (N–H), 1652 (C=O).

[15]aneN$_4$S:

^{1}H NMR (400.13 MHz; D$_2$O; DSS; pD = 1.72): δ 3.17 (t, 4H, ^3J = 6, H_a), 3.34 (s, 4H, ^3J = 6, H_e), 3.44 (t, 4H, H_d), δ 3.50 (t, 4H, ^3J = 6, H_b), 3.57 (t, 4H, ^3J = 6, H_c) ppm.

^{13}C NMR (100.61 MHz; D$_2$O; dioxane; pD = 1.72): δ 29.55 (C_a), 43.64 (C_d), 45.00 (C_c), 45.37 (C_e), 46.94 (C_b).

FT-IR (KBr, cm^{-1}):ν 3426 (N–H).

m/*z* (ESI-MS; methanol; positive ion mode) 233.20 [M + H]$^+$.

Supplementary **Table S1.** Mean ± Standard Error for each experimental group in Figures 2, 3, 4 and 5.

Figure 2a

Experimental group	Mean ± SE
Control	100
MeHg	24 ± 3.7
MeHg + [15]aneN_4S 40μM	48 ± 7.9
[15]aneN4S 40μM	97 ±6.8
MeHg + BAL 40μM	36 ± 2.8
BAL 40μM	94 ± 2.6
MeHg + DMSA 40μM	53 ± 6.3
DMSA 40μM	95 ± 6.2

Figure 2b

Experimental group	Mean ± SE
Control	100
MeHg	49 ± 6.0
MeHg + [15]aneN_4S 40μM	70 ± 2.7
[15]aneN4S 40μM	102 ±6.2
MeHg + BAL 40μM	93 ± 3.2
BAL 40μM	102 ± 3.7
MeHg + DMSA 40μM	81 ± 6.4
DMSA 40μM	93 ± 7.2

Figure 3

Experimental group	Mean ± SE
Control	100
[15]aneN_4S 10μM	96 ± 2.0
[15]aneN_4S 20μM	106 ± 11
[15]aneN4S 40μM	111 ± 4.1
[15]aneN4S 80μM	102 ± 8.1
[15]aneN4S 120μM	103 ± 14.6
BAL 10μM	91 ± 1
BAL 20μM	87 ± 4.8
BAL 40μM	87 ± 9.8
BAL 80μM	86 ± 9.1
BAL 120μM	74 ± 10.1
DMSA 10μM	100 ± 5.2
DMSA 20μM	106 ± 1.2
DMSA 40μM	108 ± 8.0
DMSA 80μM	115 ± 3.3
DMSA 120μM	115 ± 2.6

Figure 4a

Experimental group	Mean ± SE
Control	100
MeHg	33 ± 8.8
MeHg + [15]aneN_4S 40μM	36 ± 12
[15]aneN4S 40μM	111 ± 26
MeHg + BAL 40μM	35 ± 16
BAL 40μM	106 ± 16
MeHg + DMSA 40μM	28 ± 11
DMSA 40μM	82 ± 1.6

Figure 4b

Experimental group	Mean ± SE
Control	100
MeHg	47 ± 3.7
MeHg + [15]aneN_4S 40μM	105 ± 17
[15]aneN4S 40μM	102 ± 7.4
MeHg + BAL 40μM	112 ± 16
BAL 40μM	94 ± 9.4
MeHg + DMSA 40μM	48 ± 3.0
DMSA 40μM	111 ± 15

Figure 5b

Experimental group	Mean ± SE
C	1.0
M	0.87 ± 0.015
M+N	0.99 ± 0.19

N	0.99 ± 0.16
M+B	0.99 ± 0.092
B	1.0 ± 0.039
M+D	1.0 ± 0.12
D	1.02 ± 0.043

Figure 5c

Experimental group	Mean ± SE
C	1.0
M	0.88 ± 0.12
M+N	1.1 ± 0.15
N	1.0 ± 0.17
M+B	0.89 ± 0.20
B	0.77 ± 0.19
M+D	0.71 ± 0.060
D	0.69 ± 0.16
